# Immunoaffinity biosensors for deoxynivalenol determination from wheat sample: the evaluation of antibody immobilization route and surface topography on the sensor performance

**DOI:** 10.1007/s00604-026-08113-4

**Published:** 2026-05-12

**Authors:** Erdoğan Özgür, Sena Pişkin, Canan Armutcu, Mehmet Emin Çorman, Burcu Doğan-Topal, Mehmet Altay Ünal, Qingqing Yang, Lokman Uzun, Sibel A. Ozkan

**Affiliations:** 1 Department of Chemistry, Faculty of Science, Hacettepe University, Ankara, Türkiye; 2 Department of Biochemistry, Gülhane Faculty of Pharmacy, University of Health Sciences, Ankara, Türkiye; 3 Department of Analytical Chemistry, Faculty of Pharmacy, Ankara University, Ankara, Türkiye; 4 Stem Cell Institute, Ankara University, Ankara, Türkiye; 5 School of Agricultural Engineering and Food Science, Shandong University of Technology, Zibo, China

**Keywords:** Deoxynivalenol (DON), Electrochemical immunosensor, Wheat contamination, Mycotoxin detection, Food safety

## Abstract

Deoxynivalenol (DON) is one of the most common mycotoxins found in food and feed. This study aims to address the growing need for sensing platforms by applying surface engineering for the high-sensitivity detection of DON. Antibody immobilization via a self-assembled monolayer (SAM) strategy was systematically compared with immobilization on a porous poly(glycidyl methacrylate) [poly(GMA)] surface. The developed surfaces were characterized using Fourier transform infrared spectroscopy - attenuated total reflection (FTIR-ATR), atomic force microscopy (AFM), contact angle (CA) measurements, cyclic voltammetry (CV), differential pulse voltammetry (DPV), and electrochemical impedance spectroscopy (EIS). The results confirmed successful surface modification and demonstrated that surface functionalization plays a critical role in sensor performance and signal transduction. DON in standard solutions was determined using the developed AuSPE/Cys@antiDON and AuSPE/poly(GMA)@antiDON sensor platforms, with a linear working range of 2–20 ng/mL. The limit of detection (LOD) and limit of quantification (LOQ) for standard solutions were 1.43–4.33 ng/mL for AuSPE/Cys@antiDON and 1.67–5.07 ng/mL for AuSPE/poly(GMA)@antiDON, respectively. The recovery values obtained for the wheat sample were 103.72% and 99.76%, respectively. The developed sensor exhibited high selectivity, sensitivity, and repeatability performance for DON determination in both standard solutions and wheat matrix.

**Graphical Abstract:**

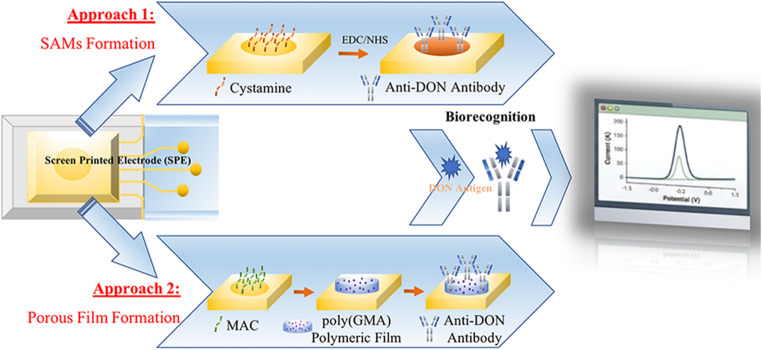

## Introduction

Grains and grain-based products constitute a major component of human and animal diets, and are highly susceptible to mold contamination. Mycotoxins can contaminate agricultural products in the field, during harvesting, or throughout storage [1], representing a major concern, particularly in rainy years, which lead to mold proliferation and enhance subsequent mycotoxin production [2–4]. Major sources of mycotoxins include cereals, cereal-based products, nuts, and spices. On the other hand, dry-cured meat products can also be contaminated through secondary contamination with mycotoxin-producing molds [5, 6]. The most common way for mycotoxins to enter the human body is through direct consumption of contaminated plant-based products. These compounds can also enter the body indirectly through the consumption of animal foods such as meat, meat products, offal, eggs, and milk due to the carryover effect from animals exposed to mycotoxins during the rearing process [7–9]. The Food and Agriculture Organization (FAO) estimates that around 25% of the world’s grain production is contaminated with mycotoxins [10]. However, as novel mycotoxins with limited or no available data are reported almost daily, their true percentage in real-world conditions is estimated to be around 50%. The most prevalent mycotoxins are predominantly *Fusarium*-derived toxins, including zearalenone, deoxynivalenol (DON), and fumonisins [11].

Deoxynivalenol (DON) is a tetracyclic epoxy-sesquiterpene belonging to the B-type trichothecene mycotoxin group [12]. The production of DON is chiefly ascribed to the molds *Fusarium graminearum* and *Fusarium culmorum*. An escalation is observed at temperatures ranging from 25 to 28 °C. This is in more humid climates characterized by water activity levels of 0.97 [13, 14]. Although DON is considered one of the least toxic trichothecenes, it remains of significant concern due to its widespread occurrence in food commodities worldwide. In humans, consuming contaminated grains can cause severe nausea, vomiting, diarrhea, abdominal pain, headache, dizziness, and fever. In animals, acute DON exposure leads to reduced feed intake (anorexia) and vomiting, while long-term exposure can result in reduced productivity, as well as pathology of the thymus, spleen, heart, and liver [15]. Synergistic interactions between DON and other mycotoxins have been reported; for example, co-exposure to aflatoxin B1 has been shown to enhance the mutagenic effects of DON [16]. Complete elimination of mycotoxins cannot be reliably achieved with conventional industrial processing methods. Therefore, ensuring food safety primarily depends on implementing controlled processing conditions and continuous monitoring throughout the production and storage stages [17–19]. The most commonly used method for screening for mycotoxins is the enzyme-linked immunosorbent assay (ELISA), which enables rapid, reproducible, and sensitive analysis of various mycotoxins. The most commonly used analytical techniques for the selective determination of mycotoxins are gas chromatography (GC), high-performance liquid chromatography (HPLC), and mass spectrometry/liquid chromatography (LC/MS) and mass spectrometry/gas chromatography (GC/MS). HPLC is the most frequently used technique for mycotoxin analyses, with fluorescent detectors (FLD) [20–22]. Since mass spectrometry provides extremely sensitive and specific detection capabilities, HPLC has increasingly been used in conjunction with mass spectrometry (MS) in the last decade [23]. The limiting factors for these techniques include the cost of equipment and the difficulty of identifying and quantifying the analyte [24]. Routine and widespread monitoring of mycotoxins is insufficient, particularly in developing countries or regions lacking centralized laboratory infrastructure. While conventional analytical methods such as LC-MS/MS, HPLC, and ELISA are reliable, they are costly and require specialized expertise, making them unsuitable for on-site analysis.

In recent years, electrochemical biosensors have been identified as a powerful alternative analytical platform for mycotoxin detection, offering low cost, short analysis times, and ease of integration into portable devices. The ability of these sensors to selectively detect specific biological recognition elements is determined by the stable immobilization of these elements on the sensor surface. In this context, antibody-based electrochemical sensors provide reliable results even in complex matrices through the high binding affinities to specific analytes.

In this study, two different surface modification strategies were developed for the electrochemical detection of deoxynivalenol (DON) in wheat matrices. The first approach was based on the formation of a self-assembled monolayer (SAM) on a gold-based screen-printed electrode (SPE) surface, providing an ordered, easy-to-prepare, and biocompatible platform for antibody immobilization. These SAM layers enabled antibodies to bind in an organized manner, protecting the active sites of the bioreceptors and improving the sensor’s reproducibility. The second approach was based on the fabrication of a porous glycidyl-functionalized polymeric film on the electrode surface via the synthesis of poly(glycidyl methacrylate) [poly(GMA)]. This porous structure enabled stable antibody immobilization via covalent bonds due to its high surface area and functional group density. Consequently, the dense, efficient placement of biorecognition elements on the sensor surface enhanced signal response and detection sensitivity. Both approaches were designed to optimize the efficiency of antibody immobilization and sensor performance. These SAM and porous polymeric film-based electrode modification strategies significantly contribute to the development of portable, highly sensitive next-generation electrochemical biosensors for the rapid and selective determination of DON in real samples.

## Experimental

### Gold SPEs (AuSPE)s modification with cystamine (AuSPE/Cys)

During the cleaning process, the gold electrodes were soaked in ethanol in an ultrasonic bath for five minutes. Then, a 0.1 M sulfuric acid (H₂SO₄) solution was used for chronoamperometric cleaning, performed at a potential of 0.9 V. The surface modification was characterized by cyclic voltammetry (CV), differential pulse voltammetry (DPV), and electrochemical impedance spectroscopy (EIS) measurements. This process was repeated until stable voltamograms were obtained for the clean electrodes. The clean electrodes were then soaked overnight in a dark environment in a 0.01 M cysteamine solution prepared in pure ethanol to form a cysteamine self-assembled monolayer (SAM) on the gold-coated surfaces. Afterward, the electrodes were washed with pure ethanol and ultrapure water, dried with argon gas, and the formed cysteamine SAMs were characterized by CV, DPV, and EIS measurements. The experimental conditions for the microscopic methods (AFM and contact angle) and FTIR-ATR were applied to characterize the electrode surface morphology and chemistry.

### Binding of biorecognition elements to the surface of SAMs (AuSPE/Cys@antiDON)

Biorecognition elements were immobilized onto the surface of modified gold electrodes via carbodiimide-mediated activation. First, 20 mg of EDC (1-ethyl-3-(3-dimethylaminopropyl) carbodiimide hydrochloride) and 5 mg of NHS (N-hydroxysuccinimide) were dissolved in a MES buffer solution at pH 5.5. Then, 100 µL of the biorecognition element (1 mg/mL of antibody in 0.1 M phosphate-buffered saline (PBS, pH 7.4) was added to the mixture and incubated at room temperature for 30 min. Then, 10 µL of the solution was dropped onto a working electrode functionalized with amino groups and incubated at 4 °C for 2 h. After the binding process, the electrodes were washed at room temperature and treated with a 1.0 M aqueous thiourea solution (pH 7.4) to passivate the surface. Following the incubation period, unbound thiourea was removed with ultrapure water. Subsequently, surface changes were characterized using CV, DPV, and EIS measurements.

### Electrode development with porous functional polymeric film

#### Synthesis of glycidyl methacrylate-based porous polymeric film (AuSPE/poly(GMA))

The synthesis of the porous polymeric film on N-methacryloyl-l-cysteine (MAC) -modified gold surface (AuSPE) was performed as follows [25]: First, a stock monomer solution was prepared containing 2-hydroxyethyl methacrylate (0.5 mL), ethylene glycol dimethacrylate (0.1 mL), deionized (DI) water (0.125 mL), and glycidyl methacrylate (GMA) (0.25 mL). An aqueous stock solution of poly(vinyl alcohol) (PVA) (25 mg/mL) was also prepared to obtain a porous structure. AIBN (a, a’-azoisobutyronitrile) (2 mg/mL), which was used as the initiator, was dissolved in dimethyl sulfoxide (DMSO). The monomer solution was prepared by mixing 100 µL of the stock monomer solution, 20 µL of the 25 mg/mL PVA stock solution, and 40 µL of the 2 mg/mL AIBN solution. Then, a 10.0 µL monomer solution was dropped onto the MAC-modified gold surface. The polymeric film was synthesized via bulk polymerization on the gold surface at 80 °C for 5 min. After the synthesis of the polymeric film, the electrode surface was rinsed with deionized (DI) water for 4 h at 25 °C under shaking conditions to remove PVA, thereby generating pores within the polymeric network. Then, anti-DON antibodies [26, 27] were immobilized on the surface. CV, EIS, and DPV measurements were performed after each modification step.

#### Covalent bonding of the biorecognition element to the polymeric film surface (AuSPE/poly(GMA)@antiDON)

The glycidyl (epoxy) groups present in the GMA monomer enable the direct immobilization of biorecognition elements, thereby eliminating the need for additional activation steps (e.g., EDC/NHS). A 1 mg/mL solution of the biological recognition element, prepared in 0.1 M PBS (pH 7.4), was applied by drop-casting onto the GMA-functionalized working electrode. The electrode was incubated overnight, then thoroughly washed at room temperature (RT) to remove unbound biomolecules. After immobilization, cyclic voltammetry (CV) and electrochemical impedance spectroscopy (EIS) were performed to monitor changes in the electrode surface’s electrochemical behavior. Surface morphology and physicochemical properties were also characterized using atomic force microscopy (AFM), contact angle measurements, and Fourier transform infrared spectroscopy - attenuated total reflection (FTIR-ATR) analysis.

### Surface characterization of electrodes

#### FTIR/ATR analysis

Surface characterization of electrodes developed for mycotoxin analysis was validated using FTIR-ATR (PerkinElmer Spectrum One, Waltham, Massachusetts, USA). The electrodes were placed in the instrument’s sample cell, and FTIR-ATR spectra in the range of 4000–400 cm^− 1^ were recorded after each modification step.

#### Atomic force microscopy analysis

The surface morphology of the anti-DON biosensor was characterized using atomic force microscopy (Nanomagnetics Instruments, Oxford, UK) in semiconductive mode. Atomic force microscopy was used to perform high-resolution measurements with a resolution of 4096 × 4096 pixels. Imaging studies were performed in air in semi-damped mode. The oscillation resonance frequency was set to 341.30 kHz. The vibration amplitude was 1 RMS, and the free vibration amplitude was 2 RMS. Samples were acquired at a scanning speed of 2 μm/s with a resolution of 256 × 256 pixels, resulting in an image of a 1 × 1 μm area.

#### Contact angle measurement

The wettability of the electrode surfaces was analyzed using water contact angle measurements with a KRUSS DSA100 instrument (Hamburg, Germany). Water droplets were applied to different regions of the electrode surface, and detailed photographs of each region were taken. The contact angles were calculated as the average of 10 measurements. The contact angle values were analyzed using DSA2 software.

#### Electrochemical characterizations

Cyclic voltammetric (voltage range: −0.2 to 0.8 V; scan rate: 50 mV/s), impedance (frequency range: 1.0 Hz to 1 kHz), and differential pulse voltammetric (voltage range: −0.2 to 0.8 V; scan rate: 50 mV/s) measurements were performed for electrochemical characterization of bare and modified electrodes. Analysis was conducted with a 5.0 mM [Fe(CN)_6_]^3−/4−^ solution as a redox probe.

### Analytical performance analysis of immunosensors

The analytical performance of the developed immunosensors was investigated using differential pulse voltammetry. DON samples prepared in both buffer solutions and wheat samples were interacted with the prepared immunosensors. The samples were prepared at concentrations of 2–20 ng/mL and analyzed using a 5.0 mM [Fe(CN)_6_]^3−/4−^ solution as a redox probe at a scanning rate of 50 mV/s and a voltage range of −0.2–0.4 V.

## Results and discussion

### Formation of cystamine-based SAMs on AuSPE (AuSPE/poly(GMA)@antiDON)

**Fig. 1:**
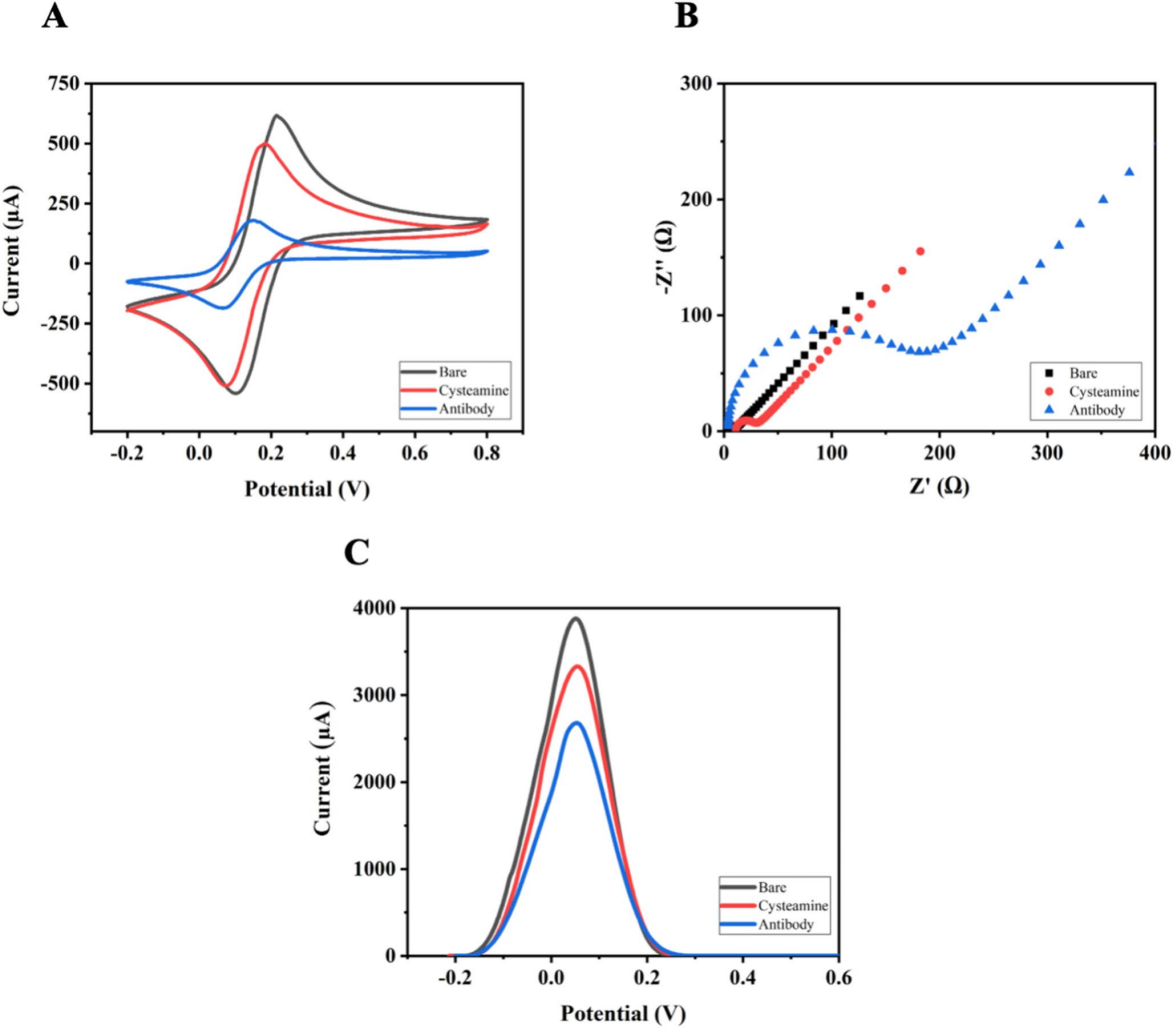
(**A**) CV, (**B**) EIS (Rct values: 12.0 Ω, 33.0 Ω, and 188.0 Ω), and (**C**) DPV curves obtained during the formation stages of the AuSPE/Cys@antiDON sensor

The electrode surface was activated with EDC/NHS to immobilize anti-DON antibodies on the surface. Anti-DON was then spotted onto the surface-modified working electrode and incubated at 4 °C for 2 h. The anti-DON-immobilized electrodes were washed at room temperature and blocked by incubation in 1.0 M thioethanol. Surface changes were characterized using CV, EIS, and DPV measurements. These methods are fundamental techniques for characterizing the conductivity of electrode surfaces, charge-transfer resistance, and electron-transfer processes. The electrochemical behavior of the electrode surfaces was evaluated using a 5.0 mM [Fe(CN)_6_]^3−/4−^ solution as a redox probe. The electrode surfaces were characterized by measuring peak currents and resistances during preparation steps, including blank AuSPE, cystamine modification, and antibody immobilization. The related CV, EIS, and DPV graphs are shown in Fig. 1A, indicating that no factors inhibited electron transfer on the blank AuSPE surface, and the peak current values of the [Fe(CN)₆]^3−/4−^ redox probe were at their maximum. After cystamine and antibody immobilization, molecules that inhibit electron transfer were deposited on the AuSPE surface, resulting in low peak current values for the [Fe(CN)_6_]^3−/4−^ redox probe. The EIS measurements evaluated the Nyquist plots and changes in load transfer resistance (Rct) **(**Fig. 1B**)**, and the same steps were applied. According to the EIS results, the blank AuSPE surface has the lowest Rct values of 12.0 Ω because electron transfer occurs easily. However, as cystamine and antibody immobilization hinder electron transfer to the AuSPE surface, the Rct values increased to 33.0 Ω after cystamine immobilization, and further to 188.0 Ω after antibody immobilization.

**Fig. 2:**
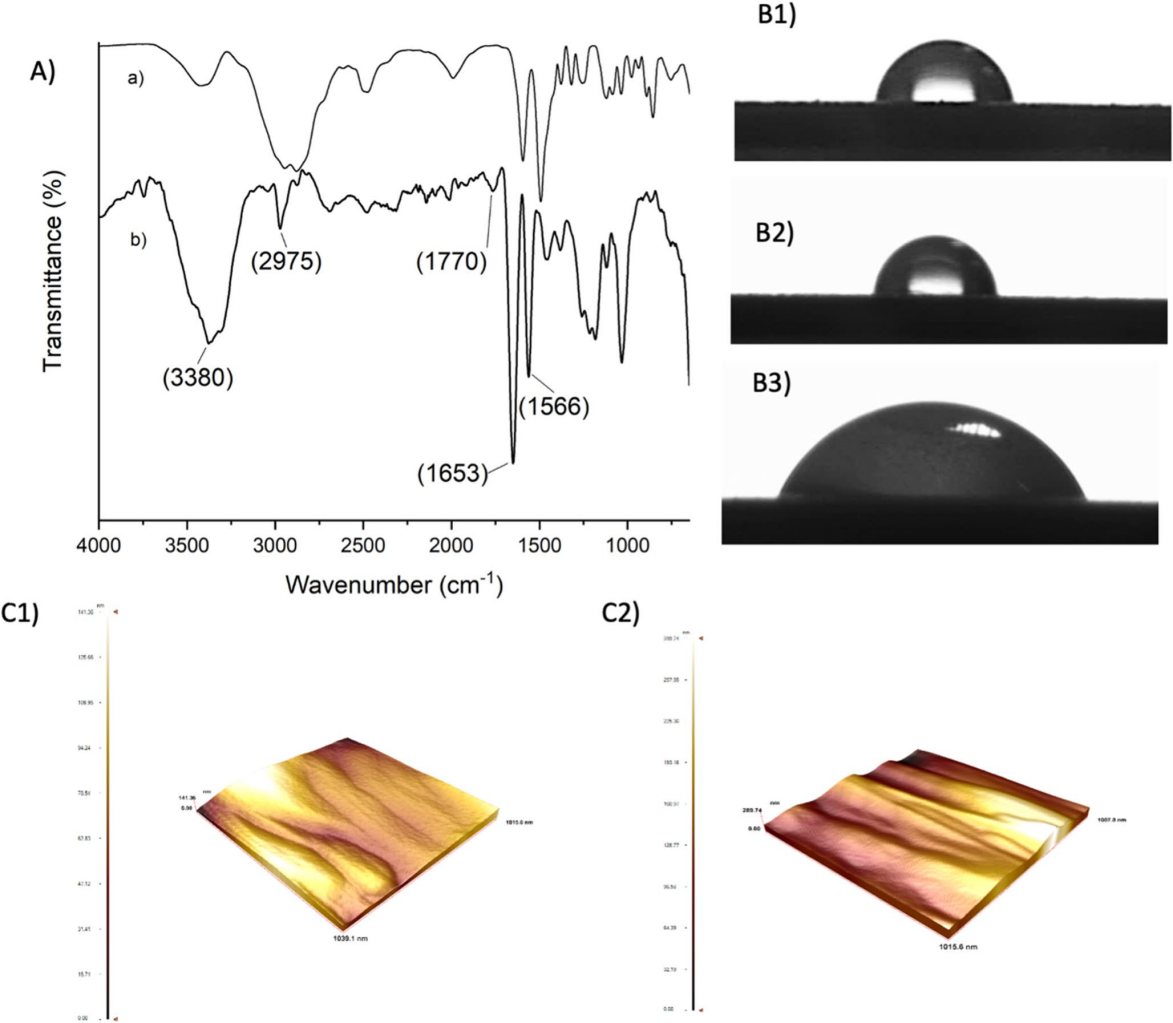
FTIR-ATR spectra of (**A**); Contact angle images of (**B1**) Blank, (**B2**) Cysteamine-modified, and (**B3**) after Anti-DON immobilization; AFM images of Cysteamine-modified, (**C1**) and after Anti-DON immobilization (**C2**) for AuSPE/Cys@antiDON

The formation of the bilayer immobilization architecture was systematically examined by FTIR spectroscopy at each step of the surface modification to confirm the successful construction of a multilayer system. Figure 2 presents the FTIR-ATR spectra corresponding to the sequential immobilization steps, specifically the cystamine monolayer (Fig. 2A) and the cystamine/anti-DON bilayer (Fig. 2B). As shown in Fig. 2A**(a)**, the FTIR-ATR spectrum of the cystamine-modified surface displays characteristic absorption bands at 1566 cm^− 1^ and 1653 cm^− 1^, which are attributed to N–H bending vibrations of primary amine groups present in the cystamine molecule. Upon immobilization of the anti-DON antibody, the FTIR-ATR spectrum of the cystamine/anti-DON bilayer **(**Fig. 2A**(b)**) reveals a new absorption band at approximately 1770 cm^− 1^, corresponding to the carbonyl (C = O) stretching vibration associated with protein structures. In addition, the broad band observed around 3380 cm^− 1^ is assigned to –OH stretching vibrations, further supporting the presence of biomolecular components on the surface.

Contact angle measurements were performed to confirm the hydrophilicity of the anti-DON immobilized sensors **(**Fig. 2B**)**. The contact angle was measured at 82.4° for a blank surface **(**Fig. 2B**1)**. The contact angle of the cystamine-based surface was 88.4°. The introduction of amine-terminated alkyl chains slightly increased the surface hydrophobicity due to the presence of nonpolar carbon backbones **(**Fig. 2B**2)**. The contact angle of the anti-DON-immobilized electrode surface was 66.4° **(**Fig. 2B**3)**. Due to its high content of hydrophilic amino acids (e.g., serine, lysine, threonine, and aspartic acid) on its surface, the antibody can form hydrogen bonds with water molecules. The anti-DON antibody was found to be more hydrophilic than the blank and cystamine-based electrodes. The decrease in contact angle following antibody immobilization indicates successful biofunctionalization of the electrode and formation of a hydrophilic biolayer, consistent with effective antibody coverage. AFM was used to evaluate the surface morphology of the AuSPE during cysteamine modification and anti-DON antibody immobilization. After anti-DON antibody immobilization, the AFM images are characterized by the appearance of heterogeneous features distributed across the surface. These features are attributed to the presence of immobilized anti-DON antibodies, forming an active layer on the Cys-modified surface. These topographical changes confirm the successful immobilization of anti-DON antibodies.

### **Synthesis of glycidyl methacrylate-based porous polymeric film on AuSPE/poly(GMA)@antiDON**

**Fig. 3:**
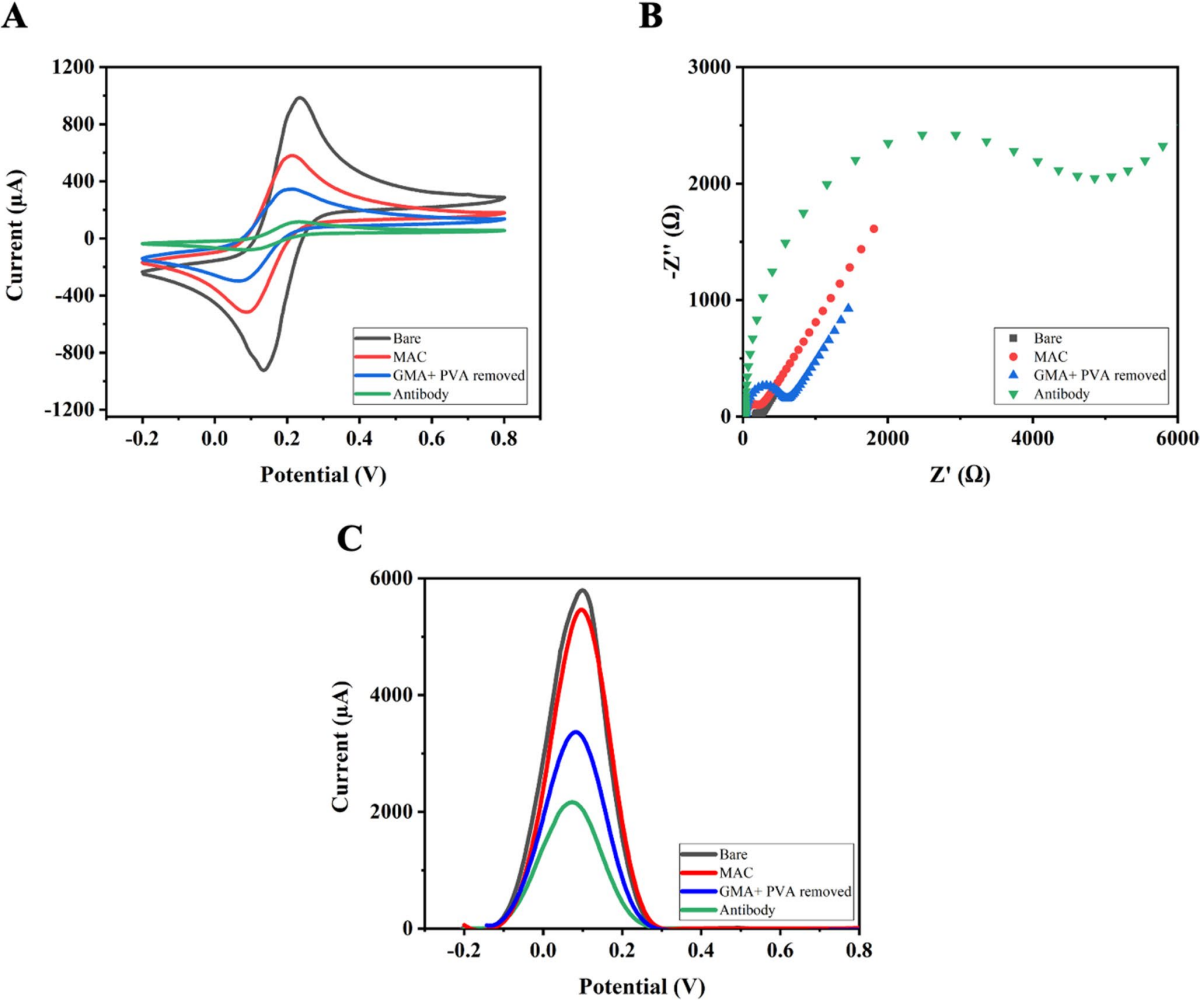
(**A**) CV, (**B**) EIS (Rct values of 25 Ω, 248 Ω, 648 Ω, and 5,084 Ω, respectively), and (**C**) DPV voltamograms recorded on AuSPE/poly(GMA)@antiDON

Figure 3A shows the cyclic voltammetry (CV) voltammograms, which indicate a decrease in peak current relative to the blank electrode, due to inhibition of electron transfer at the electrode surface after modification, polymer formation, and anti-DON immobilization. Figure 3B shows the Nyquist plots obtained from EIS measurements during the formation stages of the porous poly(GMA) biosensor. After the MAC modification process, Rct reaches 248.0 Ω, indicating initial surface activation and partial restriction of electron transfer. After poly(GMA) was added, Rct increased to 648.0 Ω due to the formation of a polymeric layer that partially hinders ionic conduction and electron mobility at the electrode-electrolyte interface. Finally, the immobilization of anti-DON yielded the highest Rct value of 5084.0 Ω, indicating the formation of a dense, insulating biolayer. **(**Fig. 3C**).** The significant increase in Rct following antibody attachment confirms the successful biofunctionalization of the electrode surface. Protein layers typically exhibit low electrical conductivity and act as effective barriers to electron transfer. Together, these findings demonstrate that each modification step effectively alters the interfacial properties, thus validating the layer-by-layer construction of the immunosensor.

**Fig. 4:**
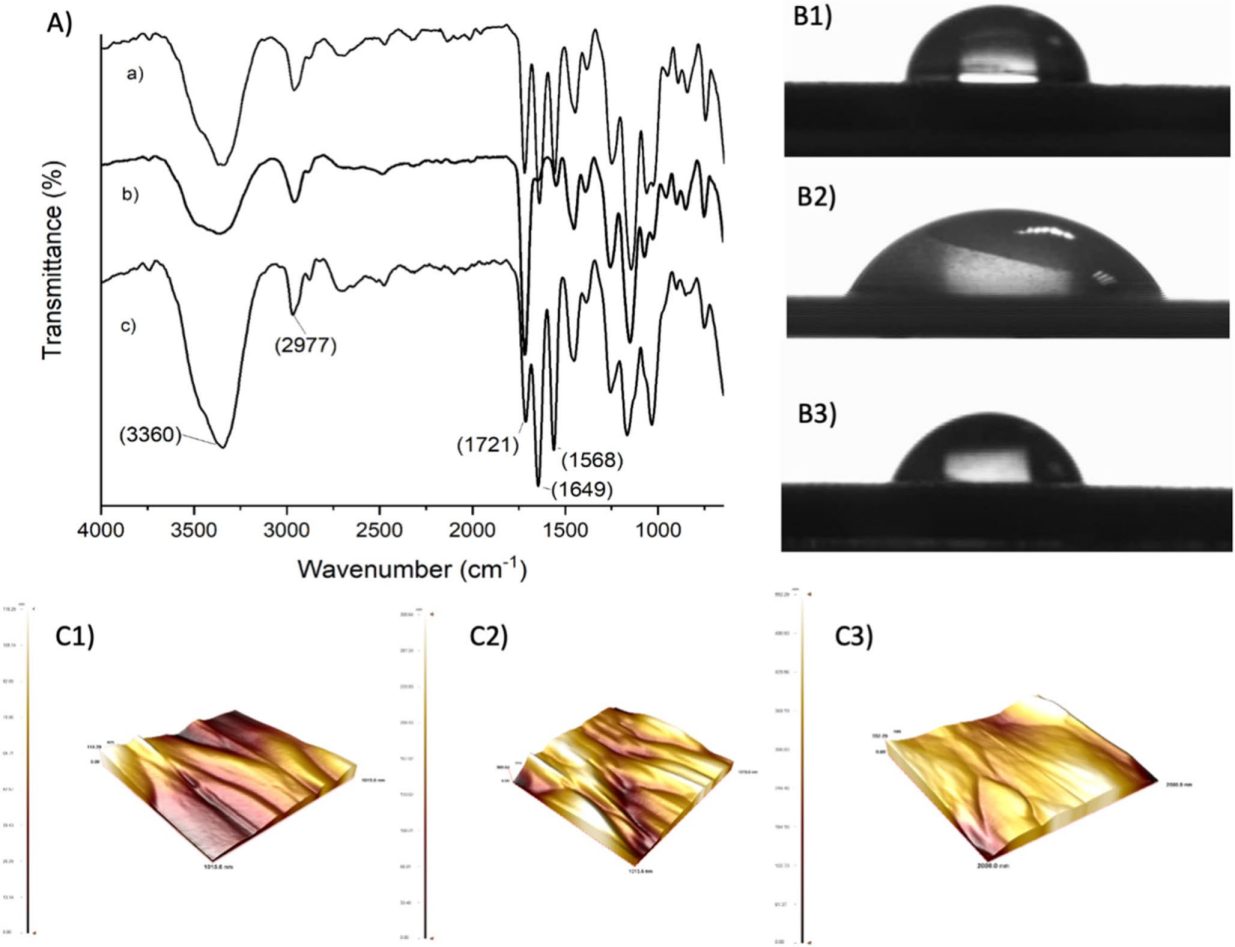
FTIR-ATR spectra of (**A**); Contact angle images of (**B1**) MAC-modified, (**B2**) after polymerization, (**B3**) anti-DON immobilized, and AFM images (**C**) of MAC-modified (**1**), poly(GMA) polymerization (**2**), and Anti-DON immobilization (**3**) for AuSPE/poly(GMA)@antiDON

Figure 4A illustrates the FTIR-ATR spectra of the MAC surface, p(HEMA-GMA), and the p(HEMA-GMA)/anti-DON modified electrode. In all spectra, a broad absorption band centered around ~ 3300 cm^− 1^ is observed, which is assigned to the overlapping stretching vibrations of –OH and –N–H functional groups, while the characteristic C–H stretching vibrations appear around 2950 cm^− 1^. Moreover, the distinct absorption band at 1717 cm^− 1^ corresponds to the C = O stretching vibration of ester groups originating from the HEMA and GMA units [28]. In addition, noticeable changes in the intensity of the absorption features within the 1500–1650 cm^− 1^ region were observed after polymerization. Upon immobilization of anti-DON antibody, the FTIR-ATR spectrum of the p(HEMA-GMA)/anti-DON electrode exhibits two newly emerged bands at ~ 1650 cm^− 1^ and ~ 1537 cm^− 1^, which are assigned to amide I (predominantly C = O stretching) and amide II (combined C–N stretching and N–H bending) vibrations characteristic of protein backbones, respectively. The appearance of these protein-related bands, together with the observed spectral changes, confirms the successful formation of the p(HEMA-GMA) polymer layer and the effective immobilization of anti-DON antibodies on the electrode surface. Examining the contact angles of the polymeric film revealed that the contact angle value of the MAC-modified surfaces was 92.6° **(**Fig. 4B**(1))**, indicating a relatively hydrophobic character due to the alkyl chain structure of MAC, which limits water molecule interaction. After polymerization, the value decreased to 66.7° **(**Fig. 4B**(2))**, reflecting an increase in surface hydrophilicity. enhances surface roughness and exposes oxygen-containing functional groups such as hydroxyl and epoxy moieties, which facilitate hydrogen bonding with water. The formation of the AuSPE surface enhances surface roughness and exposes oxygen-containing functional groups such as hydroxyl and epoxy moieties, which facilitate hydrogen bonding with water. The contact angle of the anti-DON immobilized electrode surface was 70.4° **(**Fig. 4B**(3))**. The formation of pores on the surface after polymerization, as well as the hydrophilic surface resulting from anti-DON immobilization, indicate that polymerization and immobilization were successful. The changes in contact angle at each stage of modification provide strong evidence of sequential surface modification and biofunctionalization steps, which lead to the formation of an effective sensing interface. AFM was also employed to monitor changes in surface morphology during MAC modification **(**Fig. 4C**(1))**, poly(GMA) polymerization (Fig. 4C**(2)**), and anti-DON antibody immobilization on AuSPE **(**Fig. 4C**(3))**. After anti-DON immobilization, the AFM images showed increased surface roughness. These features are attributed to the immobilized anti-DON antibodies bound to the epoxy groups of the poly(GMA) layer. The progressive topographical changes confirm successful polymer formation and antibody immobilization, resulting in an active sensing interface.

The effect of surface topography and chemistry on electron transfer and binding efficiency was analyzed by evaluating electrochemical results together with surface characterization data. Accordingly, the porous GMA-based structure offers a larger surface area for antibody binding but exhibits increased structural heterogeneity and diffusional resistance. This can be attributed to the disordered electron transfer pathways and increased impedance resulting from the three-dimensional porous architecture and is supported by EIS measurements. In contrast, SAM-modified surfaces create a regular and compact monolayer structure, allowing for more homogeneous electron transfer and enabling more controlled immobilization of antibodies [29, 30]. Therefore, SAM-based sensors offer a more uniform immobilization environment and demonstrate higher analytical performance. These findings reveal a strong relationship between surface architecture, electron transfer kinetics, and biorecognition efficiency.

### Analytical performance of Anti-DON immobilized immunosensors

**Fig. 5:**
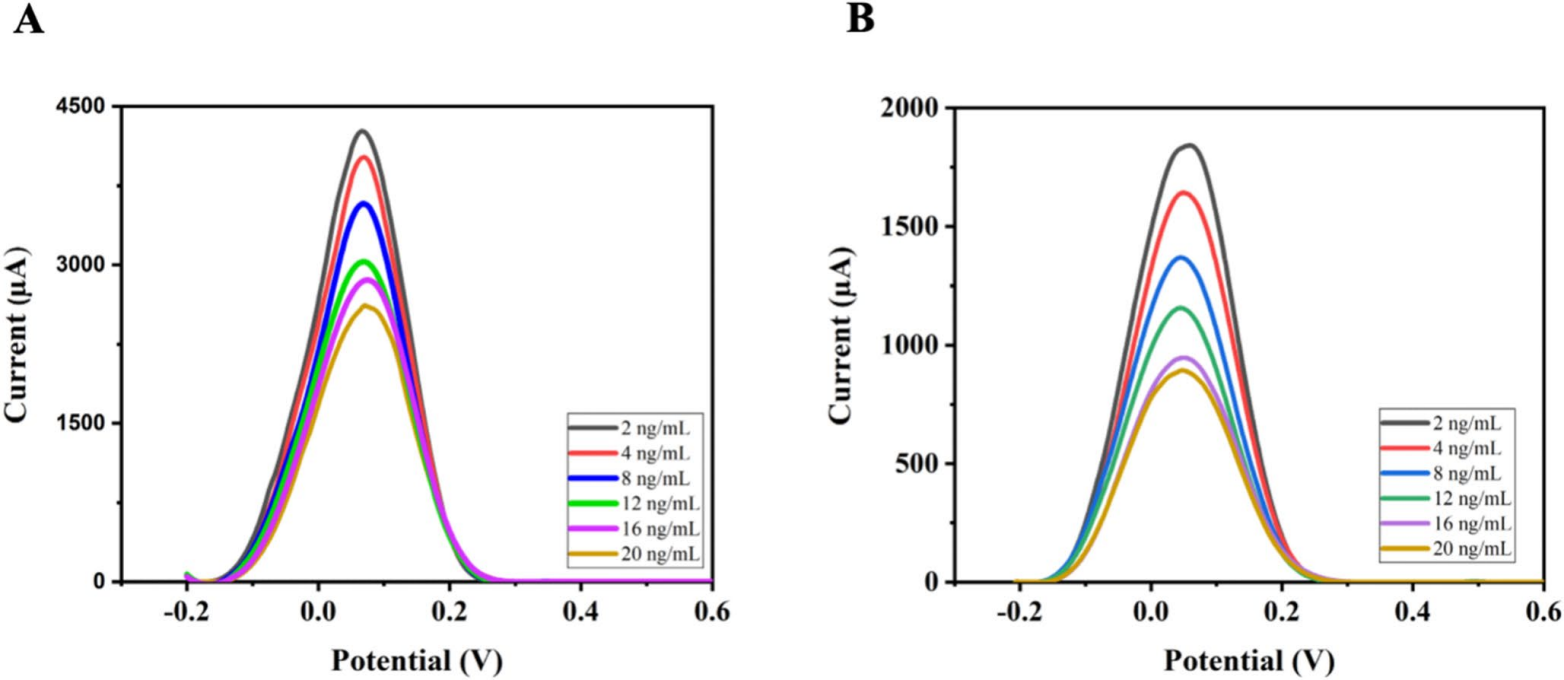
DP voltammograms corresponding to different concentrations of DON of (**A**) cystamine-based and (**B**) porous GMA-based immunoaffinity sensors

DON determination was performed using anti-DON immobilized electrochemical sensors prepared with two different approaches in the range of 2–20 ng/mL by binding different deoxynivalenol (DON) concentrations with the DPV technique **(**Fig. 5**).** 5.0 mM [Fe(CN)_6_]^3−/4−^ solution was used as the redox probe for all measurements. The corresponding calibration plots exhibited high linearity, with correlation coefficients (R²) of 0.985 for cysteamine-based self-assembled immunoaffinity sensors **(**Fig. 5A**)** and 0.980 for porous glycidyl methacrylate (GMA)-based immunoaffinity sensors **(**Fig. 5B**)**. The slightly higher correlation coefficient obtained for the cysteamine-based self-assembled monolayer (SAM) sensor indicates more homogeneous and stable immobilization of anti-DON on the gold electrode surface. In this configuration, the thiol groups of the cysteamine form strong Au–S bonds. This results in a well-oriented, densely packed monolayer that facilitates efficient electron transfer and enhances the reproducibility of binding. In contrast, the porous GMA-based structure provides a larger surface area for antibody attachment and also exhibits higher structural heterogeneity and diffusional resistance, which may result in slightly lower linearity. Therefore, SAM-based sensors provide a more controlled and uniform immobilization environment, contributing to their superior analytical performance. The results show that immunoaffinity sensors fabricated using both approaches exhibit sensitivity for DON detection (Table 1).

These two approaches have generally been studied under different conditions and mostly in buffer solutions, and studies directly comparing their behavior in complex food matrices are limited. This study offers a more holistic evaluation by considering the combined effects of surface architecture (regular SAM layer versus porous GMA-based structure) on antibody immobilization, biorecognition efficiency on the surface, and electrochemical signal response. Furthermore, the findings demonstrate a significant trade-off between the two approaches: SAM-based surfaces provide a more controlled and reproducible structure, while porous GMA-based films increase antibody loading capacity and signal intensity due to their higher surface area. This contributes to a better understanding of the relationship between the regularity of surface coating versus the density of surface functional groups in any biosensor design.

**Table 1:** Analytical performance of AuSPE/Cys@antiDON and AuSPE/poly(GMA)@antiDON immunosensors for DON determination in standard solution and wheat samples

	AuSPE/Cys@antiDON		AuSPE/poly(GMA)@antiDON	
	Standard solution	Wheat sample	Standard solution	Wheat sample
Linearity range (ng/mL)	2–20	2–20	2–20	2–20
Slope (µA/ngmL)	94.51	30.93	53.50	24.23
SE of slope	8.266	2.123	5.474	2.839
Intercept (µA)	230.91	67.64	169.3	110.2
SE of intercept	100.33	25.78	66.44	34.46
Correlation coefficient ( ***r*** )	0.985	0.990	0.980	0.973
LOD (ng/mL)	1.43	1.12	1.67	1.91
LOQ (ng/mL)	4.33	3.40	5.07	5.81

Although the linear range obtained in our study (1.43–4.33 ng/mL) appears narrower and the detection limits higher compared to some advanced sensor platforms reported in the literature, we believe that these results should be evaluated within the context of the study’s objective and application area. Firstly, considering that the maximum limits determined for DON in terms of food safety are generally at the µg/kg (ppb) level (Table 2), the sensitivity range provided by the proposed sensor platform offers sufficient sensitivity for practical detection in real samples. In this context, our study focuses on developing a reliable, reproducible, and applicable measurement platform in the real wheat matrix, rather than achieving ultra-low detection limits. Furthermore, it is known that many systems offering very low detection limits in the literature require complex nanomaterial syntheses and multi-step surface modifications. In contrast, the system developed in this study offers a portable and practical sensor design suitable for various applications with a simpler surface chemistry approach. However, the main contribution of this study is not only the analytical performance values ​​but also the comparative presentation of the effects of two different surface modification strategies (SAM and porous nanofilm) on antibody immobilization and sensor behavior. The findings provide an important insight into the relationship between surface architecture and sensitivity and selectivity.

**Table 2:** Different analytical methods for the detection of DON

Year	Sensor Type	Identification Element	Material	Linear Range	LOD	Refs
2025	Electrochemical aptasensor	Aptamer	WS₂@AuNP	0.1–500 ng/mL	0.017 ng/mL	[31]
2025	Fluorescence aptasensor	Aptamer	Magnetic beads	0.1–50 ng/mL	0.033 ng/mL	[32]
2024	MIP sensor	MIP	Mn-CeO₂ nanozyme	0.01–50 ng/mL	down to 0.003 ng/mL	[33]
2024	Biosensor	Antibody/Fab	Yeast display	0.001–132 ng/mL	0.166 pg/mL	[34]
2024	ECL aptasensor	Aptamer	Ti₃C₂ nanosheet	0.001–20 ng/mL	0.3 pg/mL	[35]
2025	Colorimetric aptasensor (nanozyme-based)	Aptamer	Pt/Au/MIL-100(Fe) nanozyme (MOF + bimetallic NP)	50–5000 ng/mL	44.14 ng/mL	[36]
2025	SERS aptasensor	Aptamer	AuNPs@Cu@C nanocomposite, Cy3 labeled aptamer	0.01–10.000 ng/mL	4.56 pg/mL	[37]
2025	Electrochemical immunosensor	Antibody	CPNM-based porous Fe-MOF nanozyme, GCE, thionine/H₂O₂	10–10^7^ pg/mL	9.6 pg/mL	[38]

### Mycotoxin analyses from wheat solutions

**Fig. 6:**
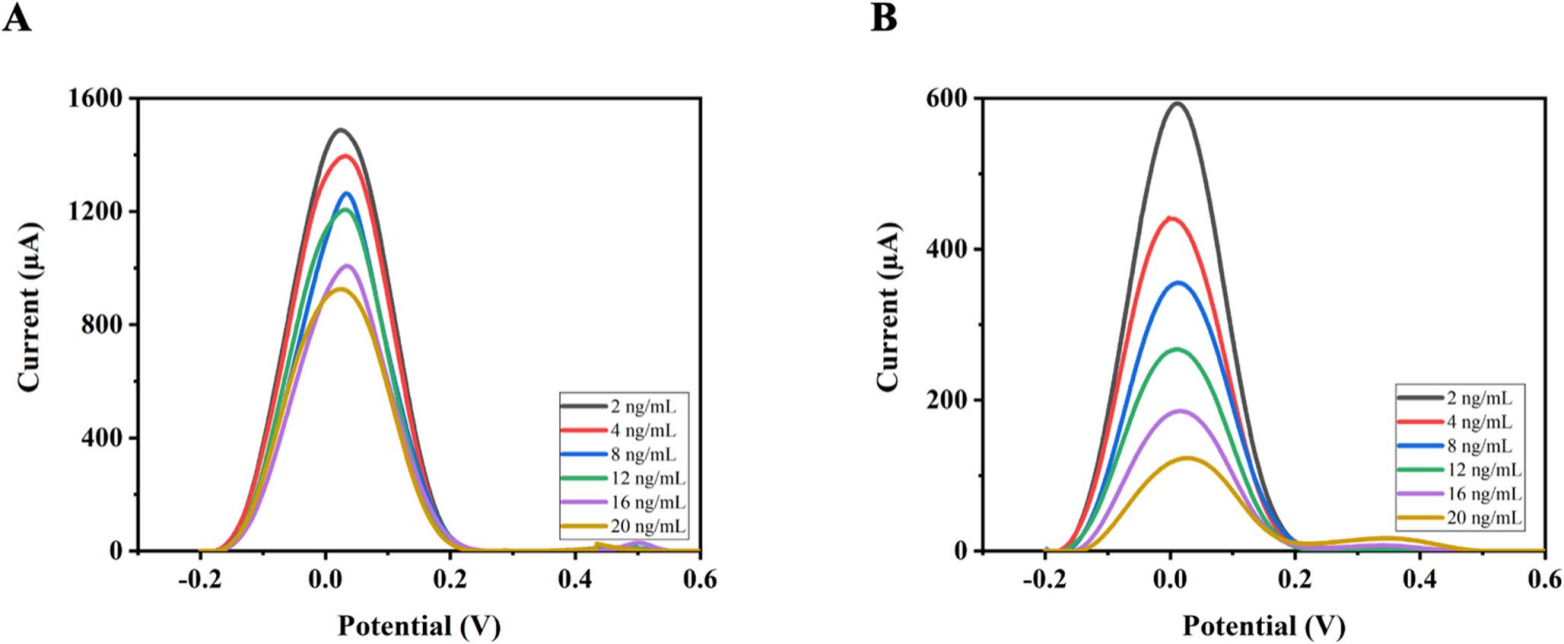
DP voltamograms and calibration curves obtained using DON antibody synthesized against different DON concentrations in the spiked wheat matrix on (**A**) cystamine-based and (**B**) porous GMA-based immunoaffinity sensors

In addition to the DON measurements performed in a buffer solution, concentration measurements were further carried out by adding DON standards to the solution extracted from a wheat grain sample. The determination of aflatoxins in wheat samples using anti-DON immobilized electrochemical sensors prepared with two different approaches was performed in the range of 2–20 ng/mL by binding different aflatoxin concentrations using the DPV technique **(**Fig. 6**)**. The calibration curve for the cystamine-based self-assembled immunoaffinity sensor exhibited R² of 0.990, whereas the porous glycidyl methacrylate (GMA)-based immunoaffinity sensors showed R² of 0.973. The results show that immunoaffinity sensors prepared by both methods have good sensitivity for aflatoxin detection (Table 3).

**Table 3:** Recovery results for DON determination in wheat samples using AuSPE/Cys@antiDON and AuSPE/poly(GMA)@antiDON immunosensors

	AuSPE/Cys@antiDON	AuSPE/poly(GMA)@antiDON
	Wheat sample	Wheat sample
Spiked amount (ng/mL)	16.0	16.0
Found amount (ng/mL)	16.60	15.96
Average recovery (%)	103.72	99.76
RSD%	1.08	1.68

It is known that aflatoxins and trichothecene derivatives can coexist in food and feed matrices, and simultaneous contamination is common. In this context, the selection of aflatoxin B1 (AFB1), aflatoxin B2 (AFB2), and aflatoxin G2 (AFG2) offers a rational approach to evaluating the performance of the developed sensor not only under model conditions but also against potential intrusions that may be encountered in complex real samples. Therefore, the use of structurally different but practically coexisting mycotoxins allows for a comprehensive evaluation of both molecular specificity and application selectivity. Therefore, to evaluate the selectivity performance of the sensor, mycotoxins with a high probability of co-occurring with the target analyte, DON, in food and feed samples were selected as interfering species. The selected mycotoxins are AFB1, AFB2, and AFG2. Although these compounds do not belong to the same chemical class as deoxynivalenol, they are critical for evaluating sensor performance due to their potential to cause contamination in real samples. This approach demonstrates not only the selectivity of the developed sensor towards the target molecule but also its analytical reliability against potential interfering species. The results regarding the selectivity performance of the sensor are shown in Table 4.

**Table 4:** Selectivity coefficients (k) calculated against DON and other mycotoxins for AuSPE/Cys@antiDON and AuSPE/poly(GMA)@antiDON surfaces

DON	k_(AuSPE/Cys@antiDON)_	k_(AuSPE/poly(GMA)@antiDON)_
	-	-
AFB1	17.21	84.86
AFB2	20.89	42.42
AFG2	9.59	66.00

## Conclusion

In this study, surface engineering was applied to ensure the sensitive determination and selectivity of DON, with the aim of detecting DON electrochemically. Characterization studies showed that the developed AuSPE/Cys@antiDON and AuSPE/poly(GMA)@antiDON sensor surfaces were successfully produced. LOD and LOQ values ​​were calculated for each surface in both standard and wheat solutions at concentrations ranging from 2.0 to 20.0 ng/mL. For the AuSPE/Cys@antiDON sensor surface, the LOD and LOQ ​​in the standard solution were 1.43 and 4.33 ng/mL, respectively, while in the wheat solution, they were 1.12 and 3.40 ng/mL. For the AuSPE/poly(GMA)@antiDON sensor surface, the LOD and LOQ ​​in the standard solution were 1.67 and 5.07 ng/mL, respectively, while in the wheat solution, they were 1.91 and 5.81 ng/mL, respectively. These two strategies both create a rational design framework for adapting electrochemical immunosensors to application-specific requirements such as sensitivity, surface stability, and fabrication complexity. More broadly, these results extend beyond DON detection and contribute to the overall development of modular electrochemical biosensor interfaces. The demonstrated compatibility of the poly(GMA) architecture with biomolecular immobilization indicates that this platform can be readily adapted for the detection of other foodborne toxins, environmental pollutants, or clinically relevant biomarkers. In the future, such surface-engineered systems may play a critical role in the transition from laboratory-based analytical methods to portable, cost-effective, and in-situ sensing technologies. In conclusion, this study provides a robust foundation for the development of next-generation electrochemical biosensors with practical applicability.

## Data Availability

No datasets were generated or analysed during the current study.
